# A scoping review examining measurement of anti-transgender stigma in low- and middle-income countries

**DOI:** 10.1371/journal.pgph.0004490

**Published:** 2025-04-30

**Authors:** Tamar Goldenberg, Amanda E. Tanner, Mohammed Sheikh Eldin Jibriel, Jennifer Toller Erausquin, Sulianie Mertus, Keenan A. Phillips, Grayson K. Rodgers, Clare Barrington

**Affiliations:** 1 Department of Public Health Education, University of North Carolina Greensboro, Greensboro, North Carolina, United States of America; 2 Department of Health Behavior, Gillings School of Global Public Health, University of North Carolina Chapel Hill, Chapel Hill, North Carolina, United States of America; Institute of Public Health Bengaluru, INDIA

## Abstract

Globally, transgender and other gender diverse (trans) people experience widespread prejudice, discrimination, violence, and other forms of stigma, which contribute to negative health outcomes. Most anti-trans stigma research has been conducted in high-income countries. Measurement of anti-trans stigma in low- and middle-income countries (LMICs) is important for understanding and improving the health of trans populations globally. Accordingly, this scoping review explores the use of quantitative anti-trans stigma measures in LMICs. This scoping review follows the guidance of the PRISMA extension for Scoping Reviews (PRISMA-ScR) Checklist and examines empirical research with trans populations in LMICs published in English, Spanish, Arabic, and Russian between 2001–2024. Study eligibility criteria included: 1) trans study population, 2) LMIC study location, 3) quantitative or mixed-method study design, and 4) quantitative measurement of anti-trans stigma. The search yielded 82 articles (representing 65 unique studies) from 34 LMICs. Most articles were published since 2018. No articles focused exclusively on trans men. About 62% of articles included a primary focus on stigma; health outcomes primarily examined HIV and mental health. Nearly all articles (95%) measured enacted stigma; other forms of stigma (e.g., internalized and anticipated) were less commonly measured, and structural stigma was only measured in 4 articles. More than half of the articles (55%) measured stigma both broadly and within specific contexts (e.g., from family, in health care). More research exploring anti-trans stigma is needed, especially with trans-masculine and other gender diverse people, measuring outcomes beyond HIV and mental health, and measuring forms of stigma beyond enacted stigma. Expanding and improving measurement of anti-trans stigma in LMICs can improve our understanding of the mechanisms shaping health equity to inform context specific and tailored health interventions to support trans communities worldwide.

## Introduction

Globally, transgender, and other gender diverse (trans) people experience widespread prejudice, discrimination, violence, and other forms of stigma [1–4]. Stigma is a fundamental cause of adverse health outcomes among trans populations as it creates chronic stress, social exclusion, and restricted access to health protective resources [4–7]. Further, stigma contributes to systemic vulnerability (e.g., unemployment, housing insecurity, limited educational opportunities, poverty), which can result in inequities across multiple health outcomes [8–12], such as HIV, violence, substance use disorders, post-traumatic stress, depression, anxiety, and suicide [3,13–16].

Measurement of anti-trans stigma in low- and middle-income countries (LMICs) is important for improving our understanding of how it shapes the health of trans populations globally. Conceptually, anti-trans stigma is the process of devaluing, threatening, and/or erasing trans identity, while reinforcing cisgender identity and the gender binary [4,5,11]. To fully understand trans individuals’ experiences with anti-trans stigma and associated health outcomes, it is critical to consider Ecological Systems Theory [17], which highlights that individual behavior and experiences are impacted by the environments in which we live. Building on Ecological Systems Theory [17], in order to understand all forms of stigma, it is important to examine anticipated, enacted, and internalized stigma, as well as stigma occurring across multiple socio-ecological levels (e.g., structural, institutional, community, interpersonal, and intrapersonal) [2,4,18,19]. Research measuring anti-trans stigma in the United States has captured some of these aspects of stigma; for example, the Gender Minority Stress scales examine multiple interpersonal and intrapersonal aspects of stigma (discrimination, rejection, victimization, non-affirmation of gender identity, internalized transphobia, negative expectations for future events, nondisclosure) and resilience (community connectedness and pride) [9]. However, a scoping review of anti-trans stigma measurement in the United States identified a gap in measuring stigma at a structural level [2].

Given the pervasiveness of anti-trans stigma [3,4] and the need for considering the varying lived experiences of trans people across countries and regions, it is important to understand how anti-trans stigma is being measured in LMICs. Yet, little research has been published on the conceptualization of anti-trans stigma, the use of anti-trans stigma measures, and the process for developing or adapting anti-trans stigma measures in LMICs. An improved understanding of the approaches that are currently being utilized for measuring anti-trans stigma across LMICs is necessary to fully understand the adversities that trans people face and to inform future research and interventions. Overall, better measurement of anti-trans stigma that considers the unique experiences of multi-marginalized communities in specific LMIC contexts can improve our understanding of the mechanisms and pathways contributing to (or hindering) health equity to inform context specific and tailored health interventions to support trans communities worldwide.

Accordingly, this scoping review explores how anti-trans stigma has been measured in LMICs, with a focus on the measures that have been used as well as the process that has been described for developing or adapting anti-trans stigma measures. These findings will inform best practices for locally conceptualized methods of measuring anti-trans stigma in LMICs.

## Materials and methods

This scoping review examines how empirical research with trans populations in LMICs has quantitatively measured anti-trans stigma. We conducted the scoping review using Arksey and O’Malley’s scoping review framework [20] and reporting follows the guidance of the PRISMA extension for Scoping Reviews (PRISMA-ScR) Checklist [21]. Methods are described in more detail in the scoping review protocol [22]. This scoping review is registered with the Open Science Framework database (osf. io/qcs2v).

### Inclusion criteria

Eligible articles included original peer-reviewed empirical articles, including both primary and secondary data analyses. Commentaries, reviews, and grey literature were excluded from the review. Based on the scoping review team’s capacity, we included articles that were published in English, Spanish, Arabic, and Russian. We considered four major areas for article eligibility and exclusion criteria, including: the study population, study location, study design, and measurement of anti-trans stigma.

#### Population.

Eligible articles needed to include trans or other gender diverse participants. Articles with both cisgender and trans participants were only included if the data were disaggregated when presenting results. We also included articles with sub-populations of trans people; for example, we included articles focused on experiences of trans women, trans men, trans people engaging in sex work, trans people living with HIV, trans youth, etc. Articles focusing on the perpetration of trans stigma among cisgender populations were not included.

#### Location.

Articles were only included if the study occurred in a LMIC, as defined by the World Bank (i.e., countries with economies that have a gross national income per capita below $14,005). Articles using data from multiple countries were included as long as at least one country was an LMIC and disaggregated data were presented for that LMIC. If the article used data from multiple countries and all countries were LMICs, we included the article even if the presentation of results was not disaggregated. Articles using data from high-income countries with immigrants from LMICs were not included.

#### Study design.

Both quantitative and mixed-methods research were included in the review if they quantitatively measured anti-trans stigma. Qualitative articles and mixed-methods articles that did not quantitatively measure anti-trans stigma were excluded.

#### Anti-trans stigma measurement.

All articles needed to include a quantitative measure of anti-trans stigma; this included articles with anti-trans stigma as a primary focus of the study, as well as those with anti-trans stigma as a descriptive sample characteristic or covariate. We applied an umbrella conceptualization of anti-trans stigma and included articles measuring anti-trans stigma in both broad and specific ways. For example, we included articles asking general questions about stigma, as well as articles examining specific dimensions of stigma or gender minority stress [9], and stigma within specific settings (e.g., discrimination within healthcare environments, anti-trans victimization or violence).

We excluded articles that exclusively measured other types of stigma (e.g., HIV stigma) and not anti-trans stigma. We also excluded articles that only measured stigma based on sexual behavior or sexual orientation, but included articles that had a combined measure for LGBTQ+ stigma that considered both sexual behavior/identity and gender identity simultaneously. Inclusion of LGBTQ+ stigma was important when trying to expand beyond a Western conceptualization of gender identity [23]; in some countries, stigma related to sexual behavior or sexual orientation may not be perceived as distinct from anti-trans stigma.

### Search strategy

We conducted an electronic database search using PubMed (Medline), WHO Global Index Medicus, and EBSCO (Health Source: Nursing/Academic Edition, LGBTQ+ Source, PsycInfo, CINAHL, and Gender Studies Database). The search was conducted in February 2024 and included articles published between January 1, 2001 and February 12, 2024.

Search terms were developed in alignment with previous scoping reviews of research with trans populations in the United States and in LMICs [2,3,24,25]. We used a combination of three search strings addressing: 1) LMICs, 2) trans identity, and 3) stigma (see S1 Table for the full list of search terms). The LMIC search string included the name of every LMIC, as well as names of regions (e.g., “Latin America”), and general terms to describe LMICs (e.g., “Global South”, “resource-limited”) to account for articles that occurred across multiple countries. The trans identity search string included a variety of general and location-specific terms (e.g., “travesti,” “kothi,” “hijira”) related to gender identity in order to include non-Western conceptualizations of trans identity. The stigma search string captured multiple dimensions of stigma (e.g., “discrim*”, “self-stigma”, “victimiz*”, “bias”). All articles included were saved in Zotero, a bibliographic software, to store and organize the manuscripts. Duplicates were removed using Zotero.

### Study selection

After removing all article duplicates, seven study team members (TG, AET, JTE, MJ, SM, KP, and GR) completed a title and abstract review; a sub-sample of abstracts were reviewed by two team members and any disagreement or questions were resolved through discussion, with decisions being made after consensus was achieved among the analysts. Next, we completed a full text review, using the same process.

### Data extraction

Seven study team members (TG, AET, JTE, MJ, SM, KP, and GR) completed the data extraction process using a data extraction table (see Tanner et al [22]) for the full data extraction table); data extraction for all articles was reviewed by at least two study team members to ensure accuracy. The review team met weekly to discuss any disagreements or questions related to data extraction and disagreements were resolved through discussion. For each included article, we recorded information about bibliographic information (authors, title, year published, journal), the study design (cross-sectional vs. longitudinal, observational vs. experimental), study location (country), study sample (description of sample, n of total sample, n of trans-specific sample), primary outcomes that were the focus of each article (including whether or not stigma was a primary focus of the article), and all stigma-related findings of each article.

The data extraction table also included information about the description of the stigma measures that were used (definition of stigma, use of a single item vs. multiple items vs. scales), information about the process for developing and/or adapting the stigma measure (if it was newly developed vs. adapted, how it was developed or adapted, and original measures used for adaptation), information about the form of stigma that was measured (e.g., internalized, anticipated, enacted, perceived, non-disclosure of identity, and structural), whether or not experiences of violence related to gender identity were included in the conceptualization of stigma, whether intersectional stigma was included as part of the stigma measure, and whether stigma was measured generally or in specific contexts. When considering the forms of stigma, we used the Health Stigma and Discrimination Framework [19], which explores both drivers and facilitators of stigma (which occur at a structural level) as well as stigma experiences (including internalized, anticipated, enacted, and perceived stigma). Internalized stigma, also referred to as self-stigma, occurs when an individual believes or endorses the negative stereotypes that society has placed on them [18,19]. Anticipated stigma refers to the expectation that stigma will occur [18,19]. Enacted stigma refers to experienced stigma, and includes experiences of discrimination, victimization, and rejection [18,19]. Perceived stigma refers to the perceptions of how a stigmatized group is treated, regardless of whether or not an individual has experienced that stigma personally [19]. Non-disclosure of identity captures the experience of concealing identity; this form of stigma is not included in the Health Stigma and Discrimination Framework, but we still included it in this scoping review because non-disclosure of identity is considered to be an aspect of gender minority stress [9]. Finally, structural stigma considers policies, cultural norms and ideologies, and other societal practices that contribute to stigma [5,8].

Specific contexts in which stigma was measured could include any specific perpetrator of stigma (e.g., police, family, healthcare workers) or setting (e.g., employment, school, health care). We assessed whether violence was included within the conceptualization of stigma because while victimization is an important aspect of stigma and minority stress [9], it can be challenging to distinguish between measuring violence in general and measuring violence specifically related to stigma.

## Results

**Fig 1:**
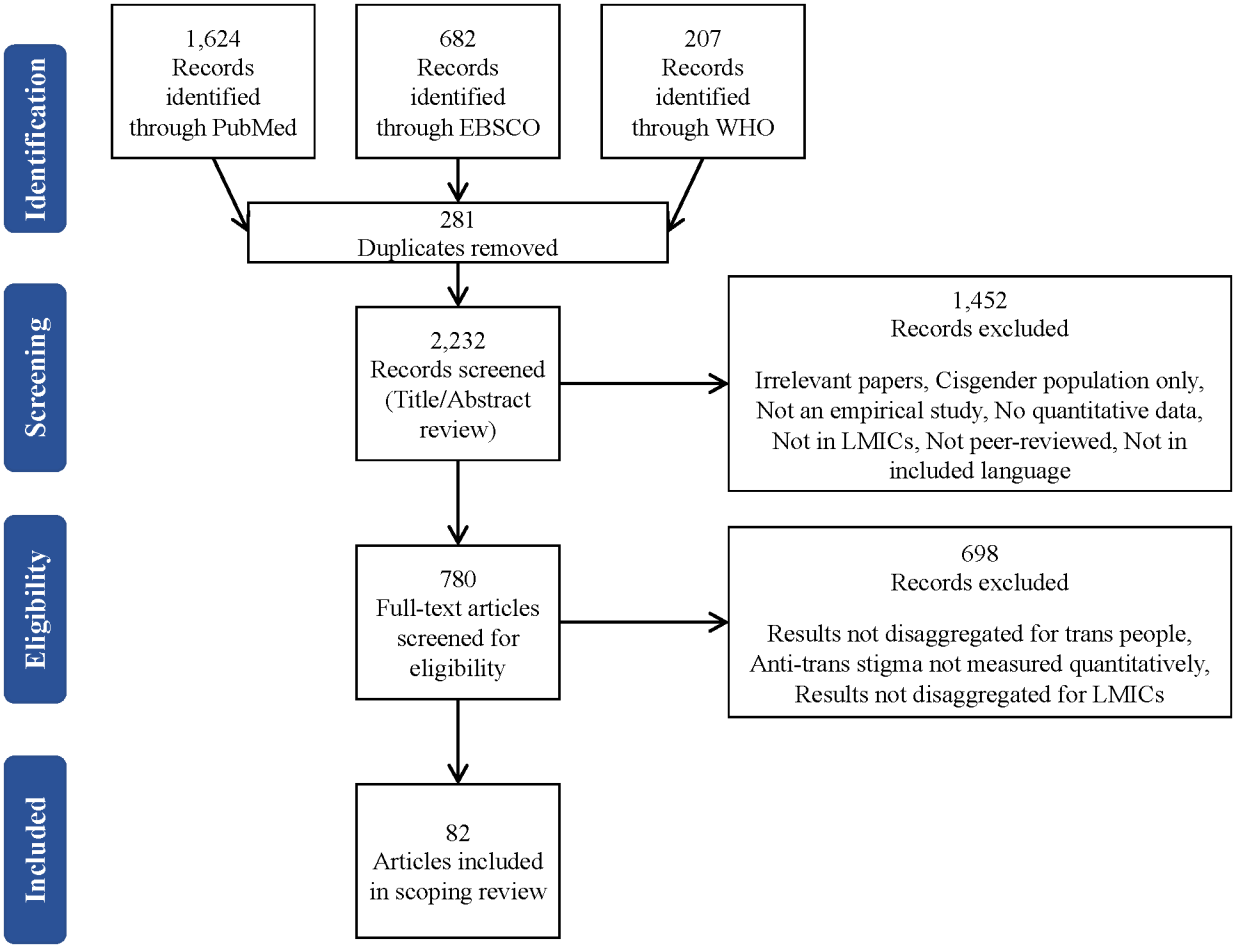
PRISMA Flow Diagram.

After removing duplicates, we screened 2,232 abstracts and completed full text review for 780 articles (Fig 1). After removing articles that failed to meet our inclusion criteria, the search yielded 82 articles (representing 65 distinct studies) from 34 LMICs [26–107], with each country having 1–14 articles; more articles took place in Brazil (n = 14 articles) and China (n = 12 articles) than any other LMIC. A list of the articles and study details is included in S2 Table. All articles were published in English except one article published in Spanish [29]. Thirty-seven (45%) articles were based in Latin America and the Caribbean, 23 (28%) in East Asia and the Pacific, ten (12%) in South Asia, six (7%) in Sub-Saharan Africa, four (5%) in Europe and Central Asia, and two (2%) in the Middle East and North Africa (See Table 1). Most LMICs did not have any articles included in this review (n = 97, 74% of LMICs).

**Table 1:** Study descriptions (n = 82 articles).

	n (%)
**Region** ^±^	
Latin America and Caribbean	37 (45%)
East Asia and Pacific	23 (28%)
South Asia	10 (12%)
Sub-Saharan Africa	6 (7%)
Europe and Central Asia	4 (5%)
Middle East and North Africa	2 (2%)
**Publication Year** ^±^	
Before 2016	3 (4%)
2016–2017	18 (22%)
2018–2019	14 (17%)
2020–2021	26 (32%)
2022–2024^*^	21 (26%)
**Trans Sample Size** ^±^	
≤100	16 (20%)
101 - 300	35 (43%)
301 - 600	16 (20%)
601 - 900	6 (7%)
901 - 1200	3 (4%)
1201 +	6 (7%)
**Participant Identity** ^+^	
General trans population	27 (33%)
Trans women/trans-feminine only	54 (66%)
Trans men/trans-masculine only	0 (0%)
Trans people engaging in sex work	7 (9%)
Trans people living with HIV	5 (6%)
Trans youth	2 (2%)
Trans parents	2 (2%)
Other specific eligibility criteria	2 (2%)
**Study Population**	
Trans Only	62 (76%)
Trans and Cis	20 (24%)
**Health Outcomes** ^+^	
HIV/STIs	37 (45%)
Mental health	27 (33%)
Stigma	19 (23%)
Substance use	13 (16%)
Violence	11 (13%)
Access to health care	10 (12%)
Hormone use	6 (7%)
Other	11 (13%)

± Percentages do not equal 100% because of rounding, but each paper is only counted one time;

+ Multiple responses possible; frequencies may not sum to 100%;

* 2024 only includes up until February 12, 2024

Most (74%, n = 61) of the included articles were published since 2018. Nearly half (43%, n = 35) had sample sizes of 101–300 participants, 27% (n = 22) had sample sizes of 301–900, and 11% (n = 9) had sample sizes greater than 900. Most articles (76%, n = 62) included only trans people in their study samples; the remainder included both trans and cisgender people (24%, n = 20). About two-thirds (66%, n = 54) of the articles only included trans women or trans-feminine participants. One-third of the articles (33%, n = 27) included a general trans population, which generally included trans-feminine, trans-masculine, and other gender diverse people. No article focused exclusively on experiences of trans-masculine or other gender diverse people. Furthermore, 9% (n = 7) of the articles included a population of trans people who engage in sex work [28,31,49,60,68,86,87], 6% (n = 5), were with trans people living with HIV [27,28,49,75,76], 2% (n = 2) were with trans youth (72,80), and 2% (n = 2) were with trans parents [88,100].

### Anti-trans stigma measures

Table 2 presents how the articles included in the scoping review measured anti-trans stigma. Among all included articles, 62% (n = 51) had a primary focus on stigma (defined as any article that included stigma as an outcome variable or a primary independent variable or framed the manuscript around the concept of stigma). In total, 6 articles (7%) included stigma as the dependent variable, 57 (70%) included stigma as an independent variable, and 3 (4%) included stigma as both a dependent and independent variable. For 16 articles this was not applicable, since they presented only descriptive statistics.

**Table 2:** Anti-trans stigma measurement information (n = 82 articles).

	n (%)
**Type of stigma measurement**+	
One single-item measure	16 (20%)
Multiple single-item measures	37 (45%)
One scale	19 (23%)
Multiple scales	18 (22%)
**Included psychometric details** (n = 37 articles that used scales)	
Yes	31 (84%)
No	6 (16%)
**Measured stigma generally or in specific contexts**	
Only measured stigma generally	10 (12%)
Only measured stigma in specific contexts (e.g., family, health care, etc.)	27 (33%)
Measured stigma generally and in specific contexts	45 (55%)
**Forms of stigma that were measured**+	
Enacted	78 (95%)
Perceived	17 (21%)
Anticipated	13 (16%)
Non-disclosure of identity	12 (15%)
Internalized	10 (12%)
Structural	4 (5%)
**Measured one vs. multiple forms of stigma** (e.g., internalized, enacted, etc.)	
Measured one form of stigma	48 (59%)
Measured multiple forms of stigma	34 (41%)
**Included violence in conceptualization of stigma measure**	
Yes	52 (63%)
No	30 (37%)

+ Multiple responses possible; frequencies may not sum to 100%

To measure anti-trans stigma, 16 articles (20%) used a single item, 37 (45%) used multiple single items (i.e., multiple questions not combined into a single score), 19 (23%) used one multi-item scale, and 18 (22%) used more than one multi-item scale. Among the 37 articles that used at least one scale, 84% (n = 31) provided psychometric properties for the scale (e.g., Cronbach’s alpha or results of factor analysis to demonstrate construct validity). Among articles that used scales and did not provide psychometrics (n = 6) [40,71,80,90,92,93], two stated that the scales had been previously identified as having good validity and/or reliability [40,92] and four made no mention of validity and/or reliability [71,80,90,93].

Fewer than half of articles (41%, n = 34) measured multiple forms of stigma. Enacted stigma was measured among nearly all the articles (95%, n = 78), with 17 articles (21%) measuring perceived stigma, 13 (16%) measuring anticipated stigma, and 10 (12%) measuring internalized stigma. In addition, 12 articles (15%) included non-disclosure of identity within their anti-trans stigma measurement and only 4 articles (5%) measured structural stigma.

Most articles measured stigma both generally and within specific settings or contexts (55%, n = 45), with 27 articles (33%) only examining stigma within specific contexts and 10 articles (12%) only examining stigma more generally (without naming any specific people or settings). Common specific contexts for measuring anti-trans stigma included family, friends, school, employment, housing, police, and health care. Most articles (63%, n = 52) included violence or victimization due to gender identity (or violence and victimization due to both gender identity and sexual orientation) within their stigma measure. While some articles mentioned intersectional stigma in the framing of anti-trans stigma, none of the anti-trans stigma measures captured intersectional stigma within quantitative study measures.

### Process of developing or adapting anti-trans stigma measures

Table 3 presents information on how the included articles described the process of developing or adapting anti-trans stigma measures. Nearly half of the articles (n = 40, 49%) provided no details about whether the anti-trans stigma measure(s) were based on existing measures, were developed for the specific study, or were adapted from previous measures. Only one article (1%) described developing a completely new anti-trans stigma measure that was not adapted from a previous measure [96]. Two articles (2%) used existing anti-trans stigma measures that had previously been used in the same specific country/regional setting [62,95]. Fifteen articles (18%) explicitly mentioned that they used an existing measure, but did not indicate whether they adapted the measure for their study. Twenty-four articles (29%) indicated that they adapted an existing measure; among these, 12 articles explicitly mentioned adaptation based on local context and 15 articles described adapting the anti-trans stigma measure from a previously existing measure that measured a different form of stigma, discrimination, violence, etc. (e.g., HIV stigma), with three articles adapting the measure based on both local context and topic [69,86,91]. Among the 15 articles that adapted their measure based on topic, seven adapted their anti-trans stigma measure based on previous measures related to sexual orientation stigma [32,51,59,60,69,81,92], four adapted their measure from HIV stigma measures [75,76,83,94], three adapted their measures from racial discrimination [54,76,86], and one article each adapted their measure based on adolescent health [91] and youth police violence [71].

**Table 3:** Process of developing or adapting anti-trans stigma measures (n = 82 articles).

	n (%)
**Developed, adapted, or created a new measure?** ^±^	
Developed a new stigma measure	1 (1%)
Adapted an existing measure^*^	24 (29%)
Used an existing measure as is (already developed for that LMIC context)	2 (2%)
Used an existing measure without indicating if adapted	15 (18%)
No mention of origin of measure	40 (49%)
**How measures were adapted** (n = 24 articles that adapted existing measures)^+^	
Existing anti-trans stigma measure adapted for the local country context	12 (50%)
Existing measure for another form of stigma adapted for anti-trans stigma	15 (58%)
**Original topics of scales adapted for anti-trans stigma** (n = 15 articles adapting for anti-trans stigma)^+^	
Sexual orientation	7 (47%)
HIV	4 (27%)
Raced-based discrimination	3 (20%)
Youth police violence	1 (7%)
Adolescent health	1 (7%)
**Adaptation/development methods** (n = 25 articles using newly developed or adapted measures)^+^	
Local expertise/community partner feedback	11 (44%)
Measure pre-testing	8 (32%)
Other	7 (28%)
No mention of adaptation/development methods	12 (48%)

± Percentages do not equal 100% because of rounding, but each paper is only counted one time;

* A paper was considered to have adapted a measure if at least one stigma measure used was adapted;

+ Multiple responses possible; frequencies may not sum to 100%

Among articles that did describe an adaptation or development process for the anti-trans stigma measures (n = 25), 11 indicated using local expertise to identify and adapt measures, eight used pre-testing of measures, and seven used other methods (e.g., cognitive interviewing, documentation of lived experiences of stigma, or modifying item wording).

### Findings from articles that explore anti-trans stigma

Overall, the articles included quantitative measures of anti-trans stigma for several purposes, including to describe how and in what setting members of trans communities experience stigma and to examine the associations between stigma and service utilization, other specific behaviors, and health outcomes, most commonly mental and sexual health. While most articles explored experiences with mental health and substance use, HIV, and health care, other health outcomes included experiences with violence, body image disturbance and disordered eating, breast/chest feeding behaviors, and parenting experiences.

#### Experiences of stigma.

Within the articles, stigma was measured in many ways including as discrimination, social exclusion, rejection, and as violence, harassment, and victimization. Overall, anti-trans stigma was commonly experienced by members of trans communities. When comparisons were made across groups, trans people reported higher levels of discrimination and violence than sexual minority cisgender participants [29,50,52,54,68,69,74,80,83,92,106].

#### Healthcare utilization.

Multiple types of healthcare use were examined, including HIV care, mental health care, gender affirming healthcare services (e.g., gender affirming hormones), and general experiences with health care. Stigma and discrimination were frequently anticipated and experienced in healthcare settings, resulting in reduced access to and uptake of healthcare services [30,44–46,48,55,84,86,87,89]. In addition, concerns related to disclosure of gender identity were found to be associated with reduced healthcare use, including mental health and HIV care [47,87]. Stigma was also associated with gender affirming hormone use [44,62,102]. Ever using gender affirming hormones was associated with higher levels of discrimination and internalized anti-trans stigma [84,103]. Trans women with experiences of gender-based sexual harassment or physical aggression were more likely to use non-prescribed gender affirming hormones [66].

#### Mental health and substance use.

Mental health outcomes were assessed in several articles (n = 30, 35%), including multiple articles examining the association of stigma with specific mental health outcomes. Anti-trans stigma was positively correlated with a variety of mental health outcomes (e.g., anxiety, depression, and suicidal ideation). More specifically, seven articles examined associations between anti-trans stigma and depression (or depressive symptoms) [39,49,63,97,102,106,107]; among those, six articles found a positive association between stigma (including discrimination and violence) and depression [39,63,97,102,106,107], and one did not find a significant association [49]. Stigma was also associated with reduced mental health service utilization [86,87], increased body image dissatisfaction [53], and increased suicide attempts [43,65,85]. Fifteen articles (17%) also described stigma and substance use behaviors. Stigma was associated with general substance use, including higher cocaine [32] and amphetamine use [67], as well as smoking cigarettes [36].

#### Sexual health.

Anti-trans stigma was associated with sexual health outcomes, mostly commonly HIV and STIs, which was included as an outcome in 29 articles (47%). Sexual risk behaviors associated with stigma varied, including engagement in sex work [33,60,81,85,96], increased reporting of sex while using substances [40,51,95], lower condom use and/or increased unprotected receptive anal intercourse [31,37,64,86,91,95], and type of male sexual partner (e.g., casual, paying, and/or multiple) [95]. Stigma also correlated with low PrEP awareness [77], willingness to use PrEP [41], and actual use [105].

In general, higher levels of stigma were related to decreases in HIV testing [58,73,103]. One article also highlighted how lower levels of internalized anti-trans stigma was associated with increased STI testing [84]. In addition, increased stigma was associated with ever being incarcerated [58] and incarceration was associated with decreased syphilis testing [61]. Articles examining the role of stigma on HIV care experiences among trans people living with HIV found associations in differing directions [28,76]. For example, one article showed that experiencing anti-trans stigma from police was associated with poorer HIV treatment outcomes, while experiencing anti-trans stigma in the workplace was associated with increased odds of receiving HIV treatment [76]. Another article found that experiencing more anti-trans stigma was associated with higher odds of viral suppression [28].

## Discussion

This is the first study to assess how anti-trans stigma is measured across LMICs. Articles were concentrated in Latin American and the Caribbean and East Asia and the Pacific. This highlights an important gap in research focused on anti-trans stigma in South Asia, sub-Saharan Africa, Europe and Central Asia, and the Middle East and North Africa. Our results about limited anti-trans research in certain global regions and contexts has implications for future research regarding anti-trans stigma in LMICs. When conducting research across diverse country settings, it is important to consider the political contexts of different countries, and the ethical considerations needed to conduct research with trans communities. While we would like to advocate for more trans-specific health research in all LMICs, we acknowledge that there are some countries where laws make research participation dangerous for trans communities; more global human rights work is needed [108].

When trying to capture the heterogeneous and multiple experiences of anti-trans stigma in LMICs, it is important to recognize that a single universal anti-trans stigma measure would likely be insufficient. Both the attributes that are stigmatized and how the stigma manifests can vary based on geographic location and cultural context. Research based in the United States underscores that the conceptualization and measurement of anti-trans stigma should be informed by lived experiences of trans people across different contexts [2]. When applying this to LMICs, this means that the measurement of anti-trans stigma may need to vary across countries or regions to account for different social, political, and cultural contexts. However, this scoping review elucidates how extant research has captured multifaceted aspects of anti-trans stigma; we identified gaps in research about and measurement of anti-trans stigma, which can help to inform future research examining anti-trans stigma in LMICs.

Our results highlight that research on anti-trans stigma in LMICs is a growing area. More than half of the articles were published since 2020, indicating that there is a recent expansion of the study of anti-trans stigma in LMICs. Overall, most articles had a primary focus on trans-feminine people. No article focused exclusively on trans-masculine or other gender diverse populations, although they were often included in articles with a general sample of trans people. These results are aligned with previous scoping reviews [24,109,110], focused on experiences of trans-masculine people in the United States and in LMICs, and highlights an important need for anti-trans stigma research to be more inclusive of the experiences of trans-masculine and other gender diverse populations. In terms of future research, much is yet to be understood about some dynamics of anti-trans stigma, and particularly the experiences of trans-masculine people.

Most of the articles that included anti-trans stigma focused on HIV, mental health, and substance use outcomes, with some research also examining healthcare use, gender affirming hormones, and experiences of violence. A few articles captured other health outcomes, including body image disturbance and disordered eating and parenting experiences, including breast/chest feeding behaviors. Given the intersecting stigmatized identities trans people may hold (e.g., gender identity, HIV status) and their disproportionate burden of negative health outcomes (e.g., HIV, depression, suicidality) [3,25,58], these foci are not surprising. However, it is important to note that these are not the only health issues that trans communities face and additional research is needed to more comprehensively understand how anti-trans stigma plays a role on other health outcomes (e.g., chronic diseases) and experiences (e.g., parenting) among trans people in LMICs. Our results underscore the need for future research to both examine and address a broader range of health outcomes.

In terms of anti-trans stigma measurement, most articles used either multiple single items or multiple scales to capture experiences of stigma, and many measured anti-trans stigma in more than one way, reflecting that current research on anti-trans stigma in LMICs is capturing multiple nuanced aspects of stigma. In addition, most articles included measures that captured both experiences with stigma more generally as well as experiences with stigma within specific contexts (e.g., family, health care, employment, police). Many of the measures also included at least one item focused on experiences with violence or victimization. However, despite using multiple items to measure stigma and despite capturing experiences of stigma broadly and within specific contexts, most articles only measured one form of stigma; enacted stigma was measured in nearly all articles. While understanding enacted stigma is important, to fully capture experiences of anti-trans stigma in LMICs, it is important to understand experiences beyond just enacted stigma [18,19], including more measures for internalized and anticipated anti-trans stigma in LMICs. Stigma frameworks and theories highlight the nuanced ways in which stigma is experienced beyond just enacted stigma [9,18,19], and therefore when measurement only focuses on enacted stigma it may not be accurately and completely measuring experiences of anti-trans stigma. Future research needs to better measure trans people’s experiences of internalized and anticipated stigma to allow for assessment of how these forms of anti-trans stigma may impact health.

Additional research that measures structural stigma is also needed, since only four articles included a measure of structural stigma. This is consistent with findings from the United States, which finds that structural anti-trans stigma is often not measured [2]. Measuring structural stigma should include a focus on policies, as well as social norms and ideologies. Structural measures of stigma should also consider how multiple and intersecting systems of oppression are reinforced. While some articles identified the importance of considering intersectional stigma and some articles measured other forms of stigma (e.g., HIV stigma, sex work stigma) in addition to anti-trans stigma, none of the articles measured intersectional stigma or used analytic methods to capture the intersectional nature of stigma. More work is needed to capture how anti-trans stigma, and especially structural forms of anti-trans stigma, functions simultaneously with other systems of oppression to influence health outcomes. It is important for future research in LMICs to prioritize the measurement of structural anti-trans stigma and intersectional stigma so that public health research, policies, and practice can better identify, understand, and resist existing power structures, which contribute to health inequities experiences by trans people [111].

Finally, most articles did not provide details about the development or adaptation of measures, which makes it difficult to assess if the measures are adequate and appropriate to capture the dimensions of stigma as experienced by trans people in a particular context. While the lack of detail provided is likely a result of limited word length when publishing findings, anti-trans stigma research would benefit from having clear and thorough explanations of how anti-trans stigma is measured in LMICs, so that measures can be applied and adapted across contexts; at minimum, it would be helpful to report on the origins of anti-trans measures and any strategies used for adapting measures. Strategies for adaptation that were identified in this review included applying local expertise of trans communities and trans-serving organizations as well as cognitive interviews and survey pre-testing. Future research in LMICs should center engagement with trans communities when developing or adapting measures of anti-trans stigma. Engagement with trans communities is important for measure development and adaptation to ensure that the measurement of anti-trans stigma is capturing the lived experiences in a specific local context; this is especially important when measuring anti-trans stigma given the nuanced and multiple ways that stigma can be defined and measured.

### Limitations

These data should be situated within the context of our scoping review. First, we were reliant on articles that identified their participants in a way we searched. We recognize the Western conceptualization of gender identity [23]; as such we included common culturally specific search terms for diverse gender identities and experiences (e.g., berdache, hijra, kothi, and waria), but we may have missed some terms. Further, in some countries, sexual and gender minorities may be perceived as part of the same group resulting in researchers not disaggregating data (providing data specific to only trans participants) or describing anti-trans stigma as stigma related to only sexual behavior/identity in quantitative stigma measures. These articles were excluded (based on exclusion criteria), thus we could both be increasing the possibility of conflating findings across these groups and/or missing country-specific nuances. While this exclusion criteria is aligned with the study goals, allowing for an understanding of broad measurements of anti-LGBTQ+ stigma, it may limit our ability to understand the ways that sexual orientation and gender identity are considered in some LMIC contexts. Furthermore, while our search included multiple databases for articles published in four languages, our search excluded research not indexed in these databases, issued in other venues (e.g., grey literature), and published in other languages (e.g., Portuguese) that would have provided a greater understanding of diversity of anti-trans stigma experiences across settings and contexts. Finally, we focused on quantitative measures of anti-trans stigma. Future research should explore the qualitative conceptualization of anti-trans stigma to fully consider the unique contexts of different LMICs.

### Conclusions

Our results highlight key areas for future research as well as specific recommendations for the measurement of anti-trans stigma in LMICs. More research is needed to fully understand the nuanced experiences of anti-trans stigma, including studies occurring in more country settings, with trans-masculine and other gender diverse people, and including more comprehensive outcomes beyond HIV and mental health [112]. In terms of measurement, research in LMICs should capture multiple forms of stigma, beyond solely focusing on enacted anti-trans stigma [18,19]. Similar to recommendations made in the United States [2], stigma measurements should consider how stigma is experienced across multiple socio-ecological levels, and especially at the structural level. In addition, more work is needed to examine anti-trans stigma using an intersectional stigma framework. Studies that are developing or adapting anti-trans stigma measures in LMICs should consider these findings and recommendations, as well as general best practices for measure development [113], but future research also needs to ensure that they include experiences and feedback from the local community when considering how to measure stigma [114–116]. Experiences of stigma vary across countries and contexts; therefore, it is necessary to understand the local experience when considering how to measure anti-trans stigma.

## Supporting information

S1 Table Search Terms for Scoping Review of Measurement of Anti-Trans Stigma in LMICs. (DOCX)

S2 Table Description of Articles Included in Scoping Review (n = 82). (DOCX)

S1 PRISMA Checklist Preferred Reporting Items for Systematic reviews and Meta-Analyses extension for Scoping Reviews (PRISMA-ScR) Checklist. (PDF)
