# Supplementary material for: A scoping review examining measurement of anti-transgender stigma in low- and middle-income countries
Source: PLOS Glob Public Health. 2025 Apr 30;5(4):e0004490. doi: 10.1371/journal.pgph.0004490 (PMC12043131; doi:10.1371/journal.pgph.0004490)
Supplement: S1 Table — (DOCX) [file pgph.0004490.s001.docx]

**Appendix 1: Search Terms for Scoping Review of Measurement of Anti-Trans Stigma in LMICs**

| **LMIC Terms** | |
| --- | --- |
| Countries | (Afghanistan[Title/Abstract] OR Albania[Title/Abstract] OR Algeria[Title/Abstract] OR Samoa[Title/Abstract] OR Angola[Title/Abstract] OR Antigua[Title/Abstract] OR Barbuda[Title/Abstract] OR Argentina[Title/Abstract] OR Armenia[Title/Abstract] OR Azerbaijan[Title/Abstract] OR Bangladesh[Title/Abstract] OR Belarus[Title/Abstract] OR Belize[Title/Abstract] OR Benin[Title/Abstract] OR Bhutan[Title/Abstract] OR Bolivia[Title/Abstract] OR Bosnia[Title/Abstract] OR Herzegovina[Title/Abstract] OR Botswana[Title/Abstract] OR Brazil[Title/Abstract] OR Bulgaria[Title/Abstract] OR “Burkina Faso”[Title/Abstract] OR Burundi[Title/Abstract] OR Cambodia[Title/Abstract] OR Cameroon[Title/Abstract] OR “Cabo Verde”[Title/Abstract] OR Central African Republic[Title/Abstract] OR Chad[Title/Abstract] OR Chile[Title/Abstract] OR China[Title/Abstract] OR Colombia[Title/Abstract] OR Comoros[Title/Abstract] OR Congo[Title/Abstract] OR “Costa Rica”[Title/Abstract] OR “Côte d'Ivoire”[Title/Abstract] OR “Cote d’Ivoire”[Title/Abstract] OR “Ivory Coast”[Title/Abstract] OR Cuba[Title/Abstract] OR Djibouti[Title/Abstract] OR Dominica[Title/Abstract] OR Dominican[Title/Abstract] OR Ecuador[Title/Abstract] OR Egypt[Title/Abstract] OR Salvador[Title/Abstract] OR Eritrea[Title/Abstract] OR Ethiopia[Title/Abstract] OR Fiji[Title/Abstract] OR Gabon[Title/Abstract] OR Gambia[Title/Abstract] OR Georgia[Title/Abstract] OR Ghana[Title/Abstract] OR Grenada[Title/Abstract] OR Guatemala[Title/Abstract] OR Guinea[Title/Abstract] OR Guinea-Bissau[Title/Abstract] OR Guyana[Title/Abstract] OR Haiti[Title/Abstract] OR Honduras[Title/Abstract] OR India[Title/Abstract] OR Indonesia[Title/Abstract] OR Iran[Title/Abstract] OR Iraq[Title/Abstract] OR Jamaica[Title/Abstract] OR Jordan[Title/Abstract] OR Kazakhstan[Title/Abstract] OR Kenya[Title/Abstract] OR Kiribati[Title/Abstract] OR Korea [Title/Abstract] OR Kosovo[Title/Abstract] OR Kyrgyz [Title/Abstract] OR Lao[Title/Abstract] OR Laos[Title/Abstract] OR Latvia[Title/Abstract] OR Lebanon[Title/Abstract] OR Lesotho[Title/Abstract] OR Liberia[Title/Abstract] OR Libya[Title/Abstract] OR Lithuania[Title/Abstract] OR Macedonia[Title/Abstract] OR Madagascar[Title/Abstract] OR Malawi[Title/Abstract] OR Malaysia[Title/Abstract] OR Maldives[Title/Abstract] OR Mali[Title/Abstract] OR Marshall[Title/Abstract] OR Mauritania[Title/Abstract] OR Mauritius[Title/Abstract] OR Mexico[Title/Abstract] OR Micronesia[Title/Abstract] OR Moldova[Title/Abstract] OR Mongolia[Title/Abstract] OR Montenegro[Title/Abstract] OR Morocco[Title/Abstract] OR Mozambique[Title/Abstract] OR Myanmar[Title/Abstract] OR Namibia[Title/Abstract] OR Nepal[Title/Abstract] OR Nicaragua[Title/Abstract] OR Niger[Title/Abstract] OR Nigeria[Title/Abstract] OR Pakistan[Title/Abstract] OR Palau[Title/Abstract] OR Panama[Title/Abstract] OR “Papua New Guinea”[Title/Abstract] OR Paraguay[Title/Abstract] OR Peru[Title/Abstract] OR Philippines[Title/Abstract] OR Romania[Title/Abstract] OR Russia[Title/Abstract] OR Russian[Title/Abstract] OR Rwanda[Title/Abstract] OR Samoa[Title/Abstract] OR “Sao Tome”[Title/Abstract] OR Senegal[Title/Abstract] OR Serbia[Title/Abstract] OR Seychelles[Title/Abstract] OR “Sierra Leone”[Title/Abstract] OR “Solomon Islands”[Title/Abstract] OR Somalia[Title/Abstract] OR “South Africa*”[Title/Abstract] OR “Sri Lanka”[Title/Abstract] OR “St. Lucia”[Title/Abstract] OR “St. Vincent”[Title/Abstract] OR Grenadines[Title/Abstract] OR Sudan[Title/Abstract] OR Suriname[Title/Abstract] OR Swaziland[Title/Abstract] OR Syria*[Title/Abstract] OR Tajikistan[Title/Abstract] OR Tanzania[Title/Abstract] OR Thailand[Title/Abstract] OR Timor-Leste[Title/Abstract] OR Togo[Title/Abstract] OR Tonga[Title/Abstract] OR Tunisia[Title/Abstract] OR Turkey[Title/Abstract] OR Turkmenistan[Title/Abstract] OR Tuvalu[Title/Abstract] OR Uganda[Title/Abstract] OR Ukraine[Title/Abstract] OR Uruguay[Title/Abstract] OR Uzbekistan[Title/Abstract] OR Vanuatu[Title/Abstract] OR Venezuela[Title/Abstract] OR Vietnam[Title/Abstract] OR “West Bank”[Title/Abstract] OR Gaza[Title/Abstract] OR Yemen[Title/Abstract] OR Zambia[Title/Abstract] OR Zimbabwe [Title/Abstract] |
| Regions | **OR** “Central Asia”[Title/Abstract] OR “Latin America”[Title/Abstract] OR “Caribbean”[Title/Abstract] OR “Middle East”[Title/Abstract] OR “North Africa”[Title/Abstract] OR “South Asia”[Title/Abstract] OR “Sub-Saharan Africa”[Title/Abstract] OR “Eastern Europe”[Title/Abstract] OR East Asia[Title/Abstract] OR Pacific[Title/Abstract] OR “South East Asia”[Title/Abstract] OR “Southeast Asia”[Title/Abstract] OR “Oceania”[Title/Abstract] |
| General terms for LMICs | **OR** “developing countries”[Title/Abstract] OR “resource-limited”[Title/Abstract] OR “resource-constrained”[Title/Abstract] OR “low- and middle-income”[Title/Abstract] OR LMIC[Title/Abstract] OR “third world”[Title/Abstract] OR “low income countries”[Title/Abstract] OR “developing nations”[Title/Abstract] OR “least developed countries”[Title/Abstract] or “less-developed countries”[Title/Abstract] OR “less-developed nations”[Title/Abstract] or “Global south”[Title/Abstract] OR “Global Health*”[MeSH] |
| **Trans Identity Terms** | |
| Trans Identity | **AND** “Transgender Persons”[MeSH] OR “Sexual and Gender Minorities”[MeSH] OR “Sex Reassignment Procedures” [Mesh] OR “Gender Identity”[MeSH] OR “Transvestism”[Mesh] OR “Transsexualism”[Mesh] OR “Health Services for Transgender Persons”[Mesh] OR transsexual*[Title/Abstract] OR transexual*[Title/Abstract] OR “trans sexual”[Title/Abstract] OR “trans-sexual*”[Title/Abstract] OR “trans-sex”[Title/Abstract] OR transgender*[Title/Abstract] OR “trans-gender*”[Title/Abstract] OR transvesti*[ Title/Abstract] OR transman[Title/Abstract] OR “trans man”[Title/Abstract] OR “transmen”[Title/Abstract] OR “trans men”[Title/Abstract] OR “transwoman”[Title/Abstract] OR “trans woman”[Title/Abstract] OR “transwomen” [Title/Abstract] OR “trans women” [Title/Abstract] OR “trans person”[Title/Abstract] OR “trans people”[Title/Abstract] OR "MTF"[Title/Abstract] OR "male to female trans*”[Title/Abstract] OR “FTM”[Title/Abstract] OR “female to male trans*”[Title/Abstract] OR transmasculin*[Title/Abstract] OR “trans masculine”[Title/Abstract] OR transfeminin*[Title/Abstract] “trans feminine” [Title/Abstract] OR “assigned female at birth”[Title/Abstract] OR “assigned male at birth”[Title/Abstract] OR “gender divers*”[Title/Abstract] OR “gender minorit*”[Title/Abstract] OR “gender affirm*”[Title/Abstract] OR agender[Title/Abstract] OR intersex[Title/Abstract] genderqueer*[Title/Abstract] OR “gender queer*”[Title/Abstract] OR genderfluid*[Title/Abstract] OR “gender fluid*”[Title/Abstract] OR “gender-fluid*”[Title/Abstract] OR “gender varian*”[Title/Abstract] OR “gender nonconform*”[Title/Abstract] OR “gender non-conform*”[Title/Abstract] OR “non-binary”[Title/Abstract] “nonbinary”[Title/Abstract] OR “gender incongruen*”[Title/Abstract] OR “gender-atypical”[Title/Abstract] OR “gender atypical*”[Title/Abstract] OR “gender identity disorder”[Title/Abstract] OR “gender dysphori*”[Title/Abstract] OR “sex reassign*”[Title/Abstract] OR “gender reassign*”[Title/Abstract] OR “gender change”[Title/Abstract] OR “sex change”[Title/Abstract] OR “gender confirmation”[Title/Abstract] OR LGBT*[Title/Abstract] OR GLBT*[Title/Abstract] OR BLGT*[Title/Abstract] OR BGLT*[Title/Abstract] OR LBGT*[Title/Abstract] OR GBLT*[Title/Abstract] OR cisgender*[Title/Abstract] OR transfeminism*[Title/Abstract] OR bigender*[Title/Abstract] OR “third gender”[Title/Abstract] OR “third sex”[Title/Abstract] OR “gender binar*”[Title/Abstract] OR “cross dress*”[Title/Abstract] OR “crossdress*”[Title/Abstract] OR “cross-dress*”[Title/Abstract] OR travesti*[Title/Abstract] OR "hijra"[Title/Abstract] OR "kothi"[Title/Abstract] OR "mahu"[Title/Abstract] OR "waria"[Title/Abstract] OR “kathoey”[Title/Abstract] OR “katoey”[Title/Abstract] OR “muxe”[Title/Abstract] |
| **Stigma Terms** | |
| Stigma | **AND**  stigma*[All Fields] OR “self stigma”[All Fields] OR “self-stigma”[All Fields] OR stereotyp*[All Fields] OR Stereotyping[MeSH] OR “shame”[All Fields] OR “shaming”[All Fields] OR “discrim*”[All Fields] OR “prejudic*”[All Fields] OR “Prejudice”[MeSH] OR “blame”[All Fields] OR “devalu*”[All Fields] OR “transphob*”[All Fields] OR “homophob*”[All Fields] OR bias*[All Fields] OR “anti-trans*”[All Fields] OR “inequalit*”[All Fields] OR “injustice*”[All Fields] OR “inequit*” [All Fields] OR “intolerance” [All Fields] OR “unfair”[All Fields] OR “minority stress*”[All Fields] OR “Social Stigma*”[MeSH] OR Labeling [MeSH] OR “Social Discrimination*”[MeSH] OR cisnorm*[All Fields] OR “reject*”[All Fields] OR “victimiz*”[All Fields] OR “violence”[All Fields] |
