# Supplementary material for: A scoping review examining measurement of anti-transgender stigma in low- and middle-income countries
Source: PLOS Glob Public Health. 2025 Apr 30;5(4):e0004490. doi: 10.1371/journal.pgph.0004490 (PMC12043131; doi:10.1371/journal.pgph.0004490)
Supplement: S2 Table — (DOCX) [file pgph.0004490.s002.docx]

**Appendix 2: Description of included articles (n=82)**

| **Authors** | **Year** | **Title** | **Country** | **Population** | **Total Sample Size** | **Trans Sample Size** | **Research Question(s)/Objective** | **Study Design** | **Forms of anti-trans stigma measured** | **Anti-trans stigma measured in specific contexts or more generally** |
| --- | --- | --- | --- | --- | --- | --- | --- | --- | --- | --- |
| Amanullah et al. | 2022 | Human rights violations and associated factors of the hijras in Bangladesh – A cross-sectional study | Bangladesh | Hijra | 346 | 346 | Examine human rights violations against hijras and assess the impact of recognizing them as third gender | Quantitative/ Cross-sectional/ Observational | Enacted, Structural | Specific and general |
| Azhar et al. | 2022 | Secret lives and gender fluidity of people living with HIV in Hyderabad, India | India | Hijra, third gender, transgender, or other gender-nonconforming (assigned male sex at birth) and living with HIV | 16 | 16 | Explore gender fluidity themes among hijra people living with HIV | Mixed Methods/ Cross-sectional/ Observational | Enacted, Perceived | Specific and general |
| Barrington et al. | 2023 | Stigma and HIV treatment outcomes among transgender women sex workers in the Dominican Republic | Dominican Republic | Transgender women engaged in sex work and living with HIV | 100 | 100 | Investigate relationship between stigma and HIV care and treatment for transgender women engaged in sex work | Quantitative/ Cross-sectional/  Observational | Anticipated, Enacted | Specific and general |
| Betancourt Llody et al. | 2019 | Situaciones que incrementan la vulnerabilidad a la infeccion por el virus de immunodeficiencia humana | Cuba | Transgender women and MSM | 522 | 58 | Describe situations contributing to HIV risk among transgender women and MSM | Quantitative/ Cross-sectional/ Observational | Enacted | Specific |
| Boonyapisomparn et al. | 2023 | Healthcare discrimination and factors associated with gender-affirming healthcare avoidance by transgender women and transgender men in Thailand: Findings from a cross-sectional online-survey study | Thailand | Transgender women and transgender men | 186 | 186 | Examine transgender women and transgender men’s attitudes and experiences with healthcare access and healthcare providers | Quantitative  Cross-sectional/ Observational | Anticipated | Specific |
| Budhwani et al. | 2017 | Transgender female sex worker's HIV knowledge, experienced stigma, and condom use in the Dominican Republic | Dominican Republic | Transgender women engaged in sex work | 78 | 78 | Examine associations between experienced stigma, HIV knowledge, and condom use among transgender women who engage in sex work | Quantitative/ Cross-sectional/ Observational | Enacted | Specific and general |
| Budhwani et al. | 2017 | Transgender women's drug use in the Dominican Republic | Dominican Republic | Transgender women | 287 | 287 | Examine associations between stigma, trauma, and drug use among transgender women | Quantitative/ Cross-sectional/ Observational | Enacted | Specific |
| Budhwani et al. | 2021 | Transgender women in Dominican Republic: HIV, stigma, substances, and sex work | Dominican Republic | Transgender women | 307 | 307 | Estimate HIV prevalence among transgender women and examine associations between stigma, sex work, substance use, and HIV among transgender women | Quantitative/ Cross-sectional/ Observational | Enacted, Perceive, Internalized | Specific |
| Campbell et al. | 2018 | Experiences of gender incongruence and the relationship between social exclusion, psychological distress, and dysfunction among South African transgender adults: A field study for ICD-11 | South Africa | Transgender adults | 57 | 57 | Describe experiences of gender incongruence among transgender adults and explored associations between social exclusion, violence, psychological distress and dysfunction during adolescence | Quantitative/ Cross-sectional/ Observational | Enacted | Specific |
| Catelan et al. | 2021 | Condom-protected sex and minority stress: Associations with condom negotiation self-efficacy, "passing" concerns, and experiences with misgendering among transgender men and women in Brazil | Brazil | Transgender men and women and travestis (characterized as trans men or women based on sex assigned at birth) | 260 | 260 | Explore associations between condom-protected sex, condom negotiation self-efficacy, self-esteem, and four dimensions of minority stress among transgender men, women and travestis | Quantitative/ Cross-sectional/ Observational | Enacted, Perceive, Anticipated, Identity concealment | Specific and general |
| Çarkaxhiu Bulut et al. | 2023 | Unveiling gender dysphoria experiences in Turkish young adults: Challenges, perspectives, and implications in health care settings | Turkey | Transgender individuals with a gender dysphoria diagnosis and cisgender individuals | 125 | 62 | Explore the formative experiences of Turkish transgender individuals to identify challenges in academic, family, and life, and solicit input on effective community interventions and treatments | Quantitative/ Cross-sectional/ Observational | Internalized, Anticipated, Enacted | Specific and general |
| Cartujano-Barrera et al. | 2023 | Prevalence and correlates of current cigarette smoking among transgender women in Argentina | Argentina | Transgender women | 393 | 393 | Assess prevalence of smoking among transgender women and examined factors associated with smoking behaviors | Quantitative/ Cross-sectional/ Observational | Enacted | Specific |
| Chakrapani et al. | 2017 | Assessment of a "transgender identity stigma" scale among trans women in India: Findings from exploratory and confirmatory factor analysis | India | Transgender women | 300 | 300 | Assess the reliability and validity of the Transgender Identity Stigma Questionnaire (TGISQ) | Quantitative/ Cross-sectional/ Observational | Enacted | Specific and general |
| Chakrapani et al. | 2017 | Understanding how sexual and gender minority stigmas influence depression among trans women and men who have sex with men in India | India | Transgender women and MSM | 600 | 300 | Examine if social support and resilient coping act as mediators or moderators on the relationship between sexual and gender minority stigmas and depression | Quantitative/ Cross-sectional/ Observational | Enacted, Perceived | Specific and general |
| Chakrapani et al. | 2019 | Syndemic classes, stigma, and sexual risk among transgender women in India | India | Transgender women | 300 | 300 | Examine associations between syndemic classes and sexual risk, determine if syndemic classes mediate effects of trans- and HIV-related stigma on sexual risk, and understand how social support and resilient coping moderate effects of syndemic classes on sexual risk | Quantitative/ Cross-sectional/ Observational | Enacted | Specific and general |
| Chakrapani et al. | 2021 | Factors influencing willingness to use human immunodeficiency virus preexposure prophylaxis among transgender women in India | India | Transgender women | 360 | 360 | Examine PrEP awareness, willingness to use PrEP, and factors that influence PrEP uptake among transgender women | Quantitative/ Cross-sectional/ Observational | Enacted | Specific |
| Chan et al. | 2024 | Gender identity differences in the experiences of family stressors and violence among transgender and non‐binary individuals in China | China | Transgender and non-binary individuals | 1063 | 1063 | Understand family stress and violence experienced by transgender and non-binary individuals in Chinese families; explore gender identity and other factors associated with family violence; and investigate the associations between family stressors, gender identity, and family violence | Quantitative/ Cross-sectional/ Observational | Enacted, non-disclosure of identity | Specific |
| Chen et al. | 2020 | A cross-sectional study of mental health, suicidal ideation and suicide attempt among transgender women in Jiangsu Province, China | China | Transgender women | 250 | 250 | Explore factors associated with mental health, suicidal ideation, and suicide attempts among transgender women in China | Quantitative/ Cross-sectional/ Observational | Enacted | General |
| Costa et al. | 2018 | Healthcare needs of and access barriers for Brazilian transgender and gender diverse people | Brazil | Transgender and gender diverse people | 626 | 626 | Assess healthcare needs and barriers experienced by transgender and other gender diverse people | Quantitative/ Cross-sectional/ Observational | Enacted | Specific |
| Durcan et al. | 2022 | Endocrinological follow-up characteristics of people diagnosed with gender dysphoria in Turkey | Turkey | Transgender men and women with gender dysphoria diagnosis | 438 | 438 | Examine loss to follow-up of transgender individuals during gender-affirming hormone therapy | Quantitative/ Longitudinal/ Observational | Enacted, Perceived | General |
| Ercan Sahin et al. | 2020 | Health status, health behaviours and healthcare access of (LGBR, gay, bisexual and transgender populations in Turkey | Turkey | Lesbian, gay, bisexual, and transgender (LGBT) people | 145 | 27 | Examine the health status, health behaviors, and healthcare access of LGBT people in Turkey | Quantitative/ Cross-sectional/ Observational | Identity concealment, Structural | Specific and general |
| Folayan et al. | 2023 | Socioeconomic inequality, health inequity and well-being of transgender people during the COVID-19 pandemic in Nigeria | Nigeria | Cisgender adolescent girls and women and transgender people living with or at high risk of acquiring HIV | 4,072 | 485 | Identify the differences in the sociodemographic factors of trans people in Nigeria compared with cisgender women living with or at elevated risk of HIV acquisition; assess the effects of the COVID-19 pandemic on access to HIV and sexual and reproductive health services; identify the differential effect of the COVID-19 pandemic on the engagement in sex work by trans and cis women; and assess the well-being of trans people during the COVID-19 pandemic | Quantitative/ Cross-sectional/ Observational | Non-disclosure of Identity | Specific |
| Garcia Ferreira et al. | 2019 | Transcendendo: A cohort study of HIV-infected and uninfected transgender women in Rio de Janeiro, Brazil | Brazil | Transgender women | 322 | 322 | Examine health factors of adult transgender women in Rio de Janerio, Brazil | Quantitative/ Longitudinal/ Observational | Enacted | Specific |
| Goldenberg et al. | 2021 | Stigma, social cohesion, and mental health among transgender women sex workers living with HIV in the Dominican Republic | Dominican Republic | Transgender women engaged in sex work and living with HIV | 100 | 100 | Examine associations between stigma, social cohesion, and mental health among transgender women engaging in sex work and living with HIV in the Dominican Republic | Quantitative/ Cross-sectional/ Observational | Enacted, Anticipated, Identity concealment | Specific and general |
| Harper et al. | 2021 | Mental health challenges and needs among sexual and gender minority people in Western Kenya | Kenya | Sexual and gender minority adults | 527 | 62 | Explore prevalence of violence, mental health, alcohol and other substance use, and differences in experiences across sexual and gender identities | Quantitative/ Cross-sectional/ Observational | Enacted | Specific and general |
| Hearld et al. | 2019 | Alcohol use, high risk behaviors, and experiences of discrimination among transgender women in the Dominican Republic | Dominican Republic | Transgender women | 291 | 291 | Examine associations between stigma, violence, alcohol use, and sex work among transgender women in the Dominican Republic | Quantitative/ Cross-sectional/ Observational | Enacted | Specific |
| Kashiha et al. | 2022 | Distinguishing trans women in men who have sex with men populations and their health access in East Africa: A Tanzanian study | Tanzania | Transgender women and MSM | 300 | 51 | Examine health care access of transgender women in seven cities in Tanzania | Quantitative/ Cross-sectional/ Observational | Enacted | General |
| Kalash et al. | 2023 | Determinants of body image disturbance and disordered eating behaviors among self-identified LGBTQ individuals | Lebanon | LGBT, queer, and heterosexual men and women | 358 | 37 | Analyze determinants of body image disturbance and disordered eating behaviors among, gay, lesbian, bisexual, transgender, and queer individuals in Lebanon | Quantitative/ Cross-sectional/ Observational | Enacted | Specific and general |
| Kranz et al. | 2023 | Minority stress among Russian lesbian, gay, bisexual, and transgender people in young and middle adulthood: Associations with internalizing and externalizing mental health problems | Russia | LGBT people in young and middle adulthood | 1,127 | 145 | Investigate group and gender differences in minority stress and mental health outcomes in Russian LGBT people and analyze associations between minority stress and mental health | Quantitative/ Cross-sectional/ Observational | Internalized, Enacted | General |
| Leite et al. | 2021 | Association between gender-based discrimination and medical visits and HIV testing in a large sample of transgender women in Northeast Brazil | Brazil | Transgender women | 864 | 864 | Examine association between gender-based discrimination, medical visits, and HIV testing among transgender women | Quantitative/ Cross-sectional/ Observational | Enacted | General |
| Leite et al. | 2022 | HIV prevalence among transgender women in Northeast Brazil – Findings from two respondent driven sampling studies | Brazil | Transgender women | 293 | 293 | Estimate the prevalence of HIV infection among transgender women and examine factors associated with HIV prevalence in Salvador, Northeast Brazil | Quantitative/ Cross-sectional/ Observational | Enacted | Specific and general |
| Lobato et al. | 2019 | Psychological distress among transgender people in Brazil: Frequency, intensity and social causation – an ICD-11 field study | Brazil | Transgender women and men | 103 | 103 | Describe experiences of gender incongruence and examine its relationships between with social rejection and psychological distress | Quantitative/ Cross-sectional/ Observational | Perceived | Specific |
| Logie et al. | 2016 | Prevalence and correlates of HIV infection and HIV testing among transgender women in Jamaica | Jamaica | Transgender women | 137 | 137 | Identify factors associated with HIV testing and HIV infection among transgender women in Jamaica | Quantitative/ Cross-sectional/ Observational | Enacted, Perceived | Specific and general |
| Logie et al. | 2017 | Associations between police harassment and HIV vulnerability among men who have sex with men and transgender women in Jamaica | Jamaica | Transgender women and MSM | 693 | 137 | Examine factors associated with police harassment of MSM and transgender women | Quantitative/ Cross-sectional/ Observational | Enacted, Perceived | Specific and general |
| Logie et al. | 2017 | Factors associated with sex work involvement among transgender women in Jamaica: A cross-sectional study | Jamaica | Transgender women | 137 | 137 | Identify social factors associated with involvement in sex work among transgender women in Jamaica | Quantitative/ Cross-sectional/ Observational | Enacted, Perceived | Specific and general |
| Logie et al. | 2018 | Factors associated with syphilis testing and a history of syphilis infection among a sample of transgender women in Jamaica | Jamaica | Transgender women | 137 | 137 | Identify factors associated with syphilis testing and a history of syphilis infection among transgender women in Jamaica | Quantitative/ Cross-sectional/ Observational | Enacted, Perceived | Specific and general |
| Lozano-Verduzco & Melendez | 2021 | Transgender individuals in Mexico: Exploring characteristics and experiences of discrimination and violence | Mexico | Transgender people | 148 | 148 | Explore experiences of discrimination and violence for transgender people in Mexico and implications on physical and mental health. | Quantitative/ Cross-sectional/ Observational | Enacted | Specific and general |
| Luz et al. | 2022 | Association of discrimination, violence, and resilience with depressive symptoms among transgender women in Rio de Janeiro, Brazil: A cross-sectional analysis | Brazil | Transgender women | 489 | 489 | Assess the relationship between resilience and past experiences of discrimination and violence with depressive symptoms among transgender women living in Rio de Janeiro | Quantitative/ Cross-sectional/ Observational | Enacted | Specific and general |
| Magno et al. | 2018 | Gender-based discrimination and unprotected receptive anal intercourse among transgender women in Brazil: A mixed methods study | Brazil | Travestis and transexual women | 127 | 127 | Examine the relationship between gender-based discrimination and unprotected receptive anal intercourse in Salvador, Brazil | Quantitative/ Cross-sectional/ Observational | Enacted | Specific |
| Marshall et al. | 2016 | Prevalence and correlates of lifetime suicide attempts among transgender persons in Argentina | Argentina | Transgender people (transwomen, transmen, or other gender) | 482 | 482 | Assess factors associated with suicide attempts among transgender people in Argentina | Quantitative/ Cross-sectional/ Observational | Enacted, Internalized | Specific and general |
| Maschiao et al. | 2020 | Nonprescribed sex hormone use among trans women: The complex interplay of public policies, social context, and discrimination | Brazil | Transgender women (travesti, mulher, transexual, transgenero) | 616 | 616 | Examine factors associated with non-prescribed hormone use among transgender women in Sao Paulo, Brazil. | Quantitative/ Cross-sectional/ Observational | Enacted | Specific and general |
| Mburu et al. | 2019 | Prevalence and correlates of amphetamine-type stimulant use among transgender women in Cambodia | Cambodia | Transgender women | 1375 | 1375 | Identify the prevalence of and factors associated with amphetamine use among transgender women in Cambodia | Quantitative/ Cross-sectional/ Observational | Enacted | Specific |
| Miller et al. | 2020 | Sex work, discrimination, drug use and violence: A pattern for HIV risk among transgender sex workers compared to MSM sex workers and other MSM in Guatemala | Guatemala | Transgender women engaged in sex work, MSM engaged in sex work, and MSM not engaged in sex work | 1057 | 122 | Examine the prevalence of and associations between alcohol and drug use, discrimination, and violence among trans women who engage in sex work compared to cis-MSM who engage in sex work and cis- MSM who do not engage in sex work in Guatemala City | Quantitative/ Cross-sectional/ Observational | Enacted | Specific and general |
| Moallef et al. | 2022 | The relationship between sexual and gender stigma and suicide attempt and ideation among LGBTQI + populations in Thailand: Findings from a national survey | Thailand | Cisgender and transgender/ intersex (including transmen, transwomen, and intersex) people | 1,290 | 647 | Assess the relationships between suicide ideation and attempt and multiple forms of sexual and gender stigma; and examine the effect of social support on the relationship between perceived and enacted dimensions of stigma and suicidal tendencies and behaviors | Quantitative/ Cross-sectional/ Observational | Enacted, Perceived | Specific and general |
| Nematollahi et al. | 2021 | Discrimination, violence, and suicide in transgender women in Iran | Iran | Transgender women | 127 | 127 | Explore the prevalence of discrimination, violence, and suicidal ideation and attempts among transgender women | Quantitative/ Cross-sectional/ Observational | Enacted | General |
| Peixoto et al. | 2021 | Interpersonal violence and passing: Results from a Brazilian trans-specific cross-sectional study | Brazil | Transgender women | 121 | 121 | Examine associations between “passing” and experiences of interpersonal violence (family and community violence) among trans women in Rio de Janeiro, Brazil | Quantitative/ Cross-sectional/ Observational | Enacted | Specific |
| Peng et al. | 2019 | Self-reported rates of abuse, neglect, and bullying experienced by transgender and gender-nonbinary adolescents in China | China | Transgender boys, girls, and non-binary individuals | 385 | 385 | Identify prevalence of violence (abuse, neglect, bullying) and examine associations between experiences of violence and mental health among transgender and non-binary students in China | Quantitative/ Cross-sectional/ Observational | Enacted | Specific |
| Pinheiro Júnior et al. | 2016 | Risk factors associated with resistance to HIV testing among transwomen in Brazil | Brazil | Transgender women | 304 | 304 | Identify factors associated with resistance to HIV testing among transgender women in Fortaleza, Brazil | Quantitative/ Cross-sectional/ Observational | Enacted | General |
| Poteat et al. | 2017 | HIV prevalence and behavioral and psychosocial factors among transgender women and cisgender men who have sex with men in 8 African countries: A cross-sectional analysis | Burkina Faso, Côte d’Ivoire, The Gambia, Lesotho, Malawi, Senegal, Swaziland, and Togo. | Transgender women and MSM | 4,586 | 937 | Estimate HIV prevalence among transgender women; identify behavioral and psychosocial HIV risk factors among transgender women and cis-MSM, and examine HIV epidemiology among transgender women compared to cis-MSM | Quantitative/ Cross-sectional/ Observational | Enacted, Perceive, Anticipated | Specific and general |
| Radusky et al. | 2020 | Reduction of gender identity stigma and improvements in mental health among transgender women initiating HIV treatment in a trans-sensitive clinic in Argentina | Argentina | Transgender women living with HIV | 61 | 61 | Describe prevalence of gender identity stigma, mental health, and substance use among transgender women during the initiation of HIV antiretroviral treatment compared to 6-months post initiation | Quantitative/ Longitudinal/ Observational | Enacted, Perceive, Internalized | Specific and general |
| Radusky et al. | 2022 | The impact of gender identity stigma and mental health on HIV treatment among transgender women in Argentina | Argentina | Transgender women living with HIV | 79 | 79 | Analyze how HIV treatment status is affected by gender identity stigma, substance use, depressive symptoms, and support from family | Quantitative/ Cross-sectional/ Observational | Internalized, Enacted | Specific and general |
| Restar et al. | 2021 | Transgender-specific developmental milestones and associated experiences of violence, discrimination, and stigma among Filipinx transgender women who are sexually active with men | Philippines | Transgender women | 139 | 139 | Describe the mean age at which gender development milestones occur and examine associations between structural violence and mean ages of gender development milestones among transgender women who have sex with men in the Philippines | Quantitative/ Cross-sectional/ Observational | Enacted, Structural | Specific and general |
| Restar et al. | 2020 | Characterizing PrEP awareness and interest among Filipina transgender women | Philippines | Transgender women | 139 | 139 | Examine PrEP awareness and interest and associations with related community-level factors among transgender women in two cities in the Philippines | Quantitative/ Cross-sectional/ Observational | Enacted, Anticipated | Specific |
| Robles et al. | 2016 | Removing transgender identity from the classification of mental disorders: A Mexican field study for ICD-11 | Mexico | Transgender people | 250 | 250 | Determine whether distress and impairment are associated with experiences of social rejection and violence rather than inherent features of transgender identity | Quantitative/ Cross-sectional/ Observational | Enacted | Specific and general |
| Ryngelblum et al. | 2023 | Violence, discrimination, and sexual health practices among adolescent men who have sex with men, transgender women and travestis in three cities in Brazil | Brazil | Adolescent MSM and adolescent transgender women (including travestis) | 884 | 72 | Evaluate how effective daily oral PrEP is in preventing HIV infection among high-risk adolescent MSM and adolescent transgender women ages 15-19 years | Quantitative/ Cross-sectional/ Intervention | Anticipated, Enacted | Specific |
| Salas-Espinoza et al. | 2017 | HIV prevalence and risk behaviors in male to female (MTF) transgender persons in Tijuana, Mexico | Mexico | Transgender women | 100 | 100 | Estimate the prevalence of HIV and examine the socio-demographics, HIV risk behaviors, and other HIV-related characteristics among transgender women in Tijuana, Mexico | Quantitative/ Cross-sectional/ Observational | Perceive, Anticipated | Specific and general |
| Santos et al. | 2021 | Travestis and transsexual women: Who are at higher risk for sexually transmitted infections? | Brazil | Transsexual women and travestis | 415 | 415 | Examine social characteristics, sexual risk behaviors, and rates of sexually transmitted infections among travestis compared to transsexual women. | Quantitative/ Cross-sectional/ Observational | Enacted | General |
| Septarini et al. | 2023 | Prevalence of stigma and discrimination amongst men who have sex with men (MSM) and transgender women (waria) in Bali, Indonesia | Indonesia | Transgender women (waria) and MSM | 416 | 123 | Examine the associations between socio-demographics and stigma and discrimination experiences for MSM and transgender women (waria) | Quantitative/ Cross-sectional/ Observational | Internalized, Anticipated,  Enacted, Non-disclosure of Identity | Specific and general |
| Sha et al. | 2021 | Gender minority stress and access to health care services among transgender women and transfeminine people: Results from a cross-sectional study in China | China | Transgender women/ trans-feminine people | 277 | 277 | Examine gender minority stress and its association to healthcare services among transgender women and trans-feminine people in China | Quantitative/ Cross-sectional/ Observational | Enacted, Internalized, Anticipated, Identity concealment | Specific and general |
| Shah et al. | 2018 | Challenges faced by marginalized communities such as transgenders in Pakistan | Pakistan | Transgender people | 189 | 189 | Examine associations between social exclusion/victimization with suicide risk, sexual behavior, and substance use among transgender people in the cities of Rawalpindi and Islamabad | Quantitative/ Cross-sectional/ Observational | Enacted | Specific and general |
| She et al. | 2021 | Impact of minority stress and poor mental health on sexual risk behaviors among transgender women sex workers in Shenyang, China | China | Transgender women engaging in sex work | 204 | 204 | Examine associations between minority stressors, poor mental health, and sexual risk behavior outcomes; and determine whether an interaction of minority stress and mental health is associated with sexual risk behavior outcomes among transgender women engaging in sex work in Shenyang, China | Quantitative/ Cross-sectional/ Observational | Enacted | Specific and general |
| She et al. | 2021 | Mental health service utilisation among transgender women sex workers who are at risk of mental health problems in Shenyang, China: An application of minority stress theory | China | Transgender women engaging in sex work | 199 | 199 | Examine prevalence of and factors associated with the use/intention to use mental health services among transgender women engaging in sex work at high risk of mental health issues in Shenyang, China | Quantitative/ Cross-sectional/ Observational | Enacted, Identity concealment | Specific and general |
| Silva et al. | 2023 | Transgender parenthood, participation in children's lives, and association with discrimination experiences: An exploratory study | Brazil | Transgender  parents and transgender non-parents | 670 | 670 (44 trans parents) | Describe the general characteristics of transgender parents, their interactions with their children, and their encounters with discrimination, in comparison to non-parent transgender individuals | Quantitative/ Cross-sectional/ Observational | Enacted, Perceived, Non-disclosure of Identity | Specific and general |
| Socias et al. | 2014 | Factors associated with healthcare avoidance among transgender women in Argentina | Argentina | Transgender women | 452 | 452 | Explore socioecological factors associated with healthcare avoidance among transgender women in Argentina | Quantitative/ Cross-sectional/ Observational | Enacted, Perceived | Specific |
| Socias et al. | 2014 | Towards full citizenship: Correlates of engagement with the gender identity law among transwomen in Argentina | Argentina | Transgender women | 452 | 452 | Explore engagement with the gender identity law among transgender women living in Argentina | Quantitative/ Cross-sectional/ Observational | Enacted, Internalized | Specific and general |
| Sohail et al. | 2022 | Frequency and risk factors associated with unprotected sex among transgenders having sex with men in Pakistan: Problem behavior theory approach | Pakistan | Hijras | 153 | 153 | Identify factors associated with unprotected sex behaviors among hijra individuals who have sex with men | Quantitative/ Cross-sectional/ Observational | Enacted, Structural | Specific and general |
| Twahira Rwema et al. | 2020 | HIV infection and engagement in HIV care cascade among men who have sex with men and transgender women in Kigali, Rwanda: A cross-sectional study | Rwanda | Transgender women and MSM | 736 | 106 | Examine HIV infection and engagement and its association with HIV care cascade among MSM and transgender women in Kigali, Rwanda | Quantitative/ Cross-sectional/ Observational | Enacted, Perceive, Anticipated | Specific |
| Wang et al | 2020 | Mapping out a spectrum of the Chinese public's discrimination toward the LGBT community: Results from a national survey | China | Heterosexual and LGBT people | 29,125 | 3,195 | Examine heterosexual participants’ attitudes towards LGBT individuals and implications for self-perceived discrimination among LGBT participants | Quantitative/ Cross-sectional/ Observational | Enacted | Specific and general |
| Weissman et al. | 2016 | HIV prevalence and risks associated with HIV infection among transgender individuals in Cambodia | Cambodia | Transgender people | 891 | 891 | Examine the prevalence of HIV, sexual risk factors, and HIV infection rate among transgender people in six cities in Cambodia | Quantitative/ Cross-sectional/ Observational | Enacted | General |
| Willie et al. | 2017 | Victimization and human immunodeficiency virus-related risk among transgender women in India: A latent profile analysis | India | Transgender women | 299 | 299 | Identify patterns of victimization among transgender women across four states in India using latent profile analysis | Quantitative/ Cross-sectional/ Observational | Enacted | Specific |
| Wilson et al. | 2021 | Population-based HIV prevalence, stigma and HIV risk among trans women in Nepal | Nepal | Transgender women (including hijra, meti, and third gender) | 200 | 200 | Estimate the HIV prevalence and examine the association between anti-trans stigma and HIV risk | Quantitative/ Cross-sectional/ Observational | Enacted, Internalized, Anticipated | Specific and general |
| Yang et al. | 2016 | A cross-sectional study of associations between casual partner, friend discrimination, social support and anxiety symptoms among Chinese transgender women | China | Transgender women | 209 | 209 | Examine the prevalence of and factors associated with anxiety symptoms among transgender women in Shenyang, China | Quantitative/ Cross-sectional/ Observational | Enacted | Specific and general |
| Yang et al. | 2015 | Sex partnership and self-efficacy influence depression in Chinese transgender women: A cross-sectional study | China | Transgender women | 209 | 209 | Examine the prevalence of depression and associated factors among transgender women in Shenyang, China | Quantitative/ Cross-sectional/ Observational | Enacted | Specific and general |
| Yang et al. | 2016 | Quality of life of transgender women from China and associated factors: A cross-sectional study | China | Transgender women | 209 | 209 | Assess the quality of life and associated factors among transgender women in Shenyang, China | Quantitative/ Cross-sectional/ Observational | Enacted | Specific and general |
| Yang et al. | 2023 | Rates of breastfeeding or chestfeeding and influencing factors among transgender and gender-diverse parents: A cross sectional study | China | Transgender and gender diverse parents | 647 | 647 | Examine breastfeeding or chestfeeding practice of transgender and gender diverse parents living in China, explore associated factors, and put forward recommendations for promoting breastfeeding or chestfeeding | Quantitative/ Cross-sectional/ Observational | Enacted | Specific |
| Yi et al. | 2020 | Social marginalization, gender-based violence, and binge drinking among transgender women in Cambodia | Cambodia | Transgender women | 1,039 | 1,039 | Examine how marginalization and gender-based violence are associated with binge drinking among transgender women in Cambodia | Quantitative/ Cross-sectional/ Observational | Enacted | Specific |
| Yi et al. | 2017 | HIV prevalence, risky behaviors, and discrimination experiences among transgender women in Cambodia: Descriptive findings from a national integrated biological and behavioral survey | Cambodia | Transgender women | 1,375 | 1,375 | Describe HIV prevalence, HIV risk behaviors, and discrimination experiences among transgender women in Cambodia | Quantitative/ Cross-sectional/ Observational | Enacted | Specific |
| Yi et al. | 2019 | Access to community-based HIV services among transgender women in Cambodia: Findings from a national survey | Cambodia | Transgender women | 1,375 | 1,375 | Identify factors associated with access to community-based HIV services for transgender women | Quantitative/ Cross-sectional/ Observational | Enacted | Specific |
| Yi et al. | 2018 | Exposure to gender-based violence and depressive symptoms among transgender women in Cambodia: Findings from the national integrated biological and behavioral survey 2016 | Cambodia | Transgender women | 1,375 | 1,375 | Examine the association between gender-based violence and depressive symptoms among transgender women in Cambodia | Quantitative/ Cross-sectional/ Observational | Enacted | Specific |
| Zalazar et al. | 2016 | High willingness to use HIV pre-exposure prophylaxis among transgender women in Argentina | Argentina | Transgender women | 337 | 337 | Assess transgender women’s willingness to use PrEP in Argentina | Quantitative/ Cross-sectional/ Observational | Enacted | Specific |
| Zea et al. | 2021 | Experiences of violence and mental health outcomes among Colombian men who have Sex with men (MSM) and transgender women | Colombia | Transgender women and MSM | 1,000 | 58 | Examine violence experiences among MSM and transgender women and the association with depressive symptoms and substance use | Quantitative/ Cross-sectional/ Observational | Enacted | General |
| Zhang et al. | 2021 | The buffer of resilience in the relations of gender-related discrimination, rejection, and victimization with depression among Chinese transgender and gender non-conforming individuals | China | Transgender and gender nonconforming individuals | 361 | 361 | Examine associations between gender minority stressors and depression; and determine if resilience moderates the relationship between gender minority stressors and depression | Quantitative/ Cross-sectional/ Observational | Enacted | Specific and general |
